# Prognostic Implication and Oncogenic Role of PNPO in Pan-Cancer

**DOI:** 10.3389/fcell.2021.763674

**Published:** 2022-01-21

**Authors:** Lingyun Zhang, Xin Li, Jinguo Zhang, Guoxiong Xu

**Affiliations:** ^1^ Department of Medical Oncology, Zhongshan Hospital, Fudan University, Shanghai, China; ^2^ Cancer Center, Zhongshan Hospital, Fudan University, Shanghai, China; ^3^ Center of Evidence-Based Medicine, Fudan University, Shanghai, China; ^4^ Research Center for Clinical Medicine, Jinshan Hospital, Fudan University, Shanghai, China; ^5^ Department of Oncology, Shanghai Medical College, Fudan University, Shanghai, China; ^6^ Department of Medical Oncology, The First Affiliated Hospital of University of Science and Technology of China, Hefei, China

**Keywords:** drug sensitivity, immune, multi-analyses, prognosis, pyridoxine 5′-phosphate oxidase, tumorigenesis

## Abstract

**Objective:** Pyridoxine 5′-phosphate oxidase (PNPO) is a key enzyme in the metabolism of vitamin B6 and affects the tumorigenesis of ovarian and breast cancers. However, the roles of PNPO in other types of cancer remain unknown.

**Methods:** The expression of PNPO was interpreted by The Cancer Genome Atlas (TCGA) database and Genotype Tissue-Expression (GTEX) database. Analysis of PNPO genomic alterations and protein expression in human organic tissues was analyzed by the cBioPortal database and human multiple organ tissue arrays. PNPO with drug sensitivity analysis was performed from the CellMiner database. The correlations between PNPO expression and survival outcomes, clinical features, DNA mismatch repair system (MMR), microsatellite instability (MSI), tumor mutation burden (TMB), and immune-associated cell infiltration were analyzed using the TCGA, ESTIMATE algorithm, and TIMER databases. Gene Set Enrichment Analysis (GSEA) was applied to elucidate the biological function of PNPO in pan-cancer.

**Results:** The differential analysis showed that the level of PNPO mRNA expression was upregulated in 21 tumor types compared with normal tissues, which was consistent with its protein expression in most cancer types. The abnormal expression of PNPO could predict the survival outcome of patients with esophageal carcinoma (ESCA), kidney renal clear cell carcinoma (KIRC), prostate adenocarcinoma (PRAD), ovarian serous cystadenocarcinoma (OV), and uveal melanoma (UVM). Furthermore, the most frequent mutation type of PNPO genomic was amplified. Moreover, the aberrant PNPO expression was related to MMR, MSI, TMB, and drug sensitivity in various types of cancer. The expression of PNPO was related to the infiltration levels of various immune-associated cells in pan-cancer by ESTIMATE algorithm and TIMER database mining.

**Conclusion:** Our results suggest that PNPO is a potential molecular biomarker for predicting patient prognosis, drug sensitivity, and immunoreaction in pan-cancer.

## Introduction

Pyridoxine 5′-phosphate oxidase (PNPO) is a key enzyme in the metabolism of vitamin B6 and could convert pyridoxine 5′-phosphate and pyridoxamine 5′-phosphate into pyridoxal 5′-phosphate (PLP) (di Salvo et al., 2011). PLP is an essential co-factor required for more than 140 enzymes and the active form of vitamin B6 (Lumeng et al., 1974; Ink and Henderson, 1984). Previous reports showed that PLP expression level was significantly related to cancer risk (Mocellin et al., 2017), while it also participates in many courses of metabolism including the metabolic process of proteins, carbohydrates, and fats (Frey and Reed, 2011). Thus, PNPO, a rate-limiting enzyme to regulate the production of PLP, could also take an important effect on these metabolisms (Ngo et al., 1998; di Salvo et al., 2011). PNPO has been known to play a part in human epilepsy (Hoffmann et al., 2007; Khayat et al., 2008; Veeravigrom et al., 2015), and some research displayed that PNPO has played a role in the development of ovarian, breast, and colorectal cancers (Chen et al., 2017; Zhang et al., 2017; Ren et al., 2019). It could regulate the biological behavior in human ovarian and breast cancer cells, indicating that PNPO may be a vital factor in tumorigenesis (Zhang et al., 2017; Ren et al., 2019). PNPO was reported as an oncogene to promote cell proliferation, migration, and invasion, and regulate cell cycle and apoptosis in ovarian and breast cancer cell lines (Zhang et al., 2017; Ren et al., 2019). PNPO combined with other six genes has been proven to be an independent prognostic index panel for OS in patients with colorectal cancer (Chen et al., 2017). However, there is a lack of research on PNPO and other cancers until now.

Recently, many studies were interested in the analysis of tumorigenesis and progression of pan-cancer, which could reveal the similarities and differences in cancers. For example, as we know, HER2 and HER3 played vital functions in the development of HER2-amplified breast cancer. The expression and constitutive phosphorylation of HER3 were reported to be ubiquitous in HER2-amplified breast cancer cell lines. However, advanced data showed that the role of HER3 was different in other HER2-amplified cancer types, suggesting that its role was variable and dependent on the disease context (Majumder et al., 2021). Thus, it is meaningful to further explore the spectrum of an oncogene in pan-cancer. So far, there is still no associated analysis between PNPO and pan-cancer. We performed systemic research on the roles of PNPO in human pan-cancer. We comprehensively investigated the different expression levels of PNPO in pan-cancer and normal control tissues *via* The Cancer Genome Atlas (TCGA), Genotype-Tissue Expression (GTEX), and Oncomine databases. Meanwhile, the prognostic value of PNPO to predict survival outcomes was also evaluated. Then, we searched for the potential relationship between PNPO mRNA expression level and clinical features, DNA mismatch repair system (MMR), microsatellite instability (MSI), tumor mutation burden (TMB), and infiltrating immune cells in pan-cancer. PNPO genomic alternations were also determined using the cBioPortal database. Furthermore, the Gene Set Enrichment Analysis (GSEA) was applied to elucidate the biological function of PNPO in pan-cancer. Drug sensitivity analysis of PNPO was performed by the CellMiner™ database. In summary, our results indicated that PNPO could serve as a candidate prognostic factor among a variety of cancers. PNPO exerts its function *via* the regulation of MMR, MSI, TMB, tumor immune microenvironment, and drug sensitivity. This study highlights the multifaceted role of PNPO in pan-cancer, which provides a rationale for targeting PNPO as a novel therapeutic strategy.

## Materials and Methods

### Data Processing and PNPO Expression Analysis

The UCSC Xena (https://xena.ucsc.edu/) was used to examine the transcriptome data of pan-cancer and clinical characters in TCGA. Human normal tissue expression matrices were obtained from the GTex Portal (https://www.gtexportal.org/). The Strawberry Perl scripts software (Version 5.30.0.1, http://strawberryperl.com/) was performed to extract the PNPO expression data in 33 TCGA tumor types and GTex normal tissues. PNPO expression was also evaluated in various tumor types from the Oncomine database (www.oncomine.org) (Rhodes et al., 2007). PNPO mRNA expression levels in healthy men and women tissues were observed by the “gganatogram” R package. The expression data were transformed with log2 (TPM) excluding missing data and duplicated values. The analysis was done by R version 4.0.2 software (https://www.Rproject.org). PNPO mRNA expression in cell lines was obtained using Cancer Cell Line Encyclopedia (https://portals.broadinstitute.org/ccle/) (Barretina et al., 2012).

### Tissue Microarray

Human multiple organ tissue arrays (Cat# HOrgC110PT01 and HOrgC120PG05, Shanghai Outdo Biotech Co., Ltd., Shanghai, China) were used to test the protein expression of PNPO by immunohistochemistry. The study of the human subject was approved by the Ethics Committee of Shanghai Outdo Biotech Co., Ltd. A total of 230 paraffin-embedded tissue samples were collected, and of these, 2 samples were either invalid or missing. All the other 228 samples were valid, including 121 malignant tumor tissues, 89 adjacent tissues of cancer, and 18 normal tissues (Supplementary Table S1). The anti-PNPO antibody (1:2000 dilution, #15552-1-AP) was obtained from ProteinTech Group (Chicago, IL, United States).

### Correlation of PNPO Expression With Survival Prognosis and Clinical Features

Cox proportional hazard models and Kaplan–Meier plotter analysis were used to assess the relationship between PNPO mRNA expression and survival outcomes including overall survival (OS), progression-free interval (PFI), disease-free interval (DFI), and disease-specific survival (DSS). The “survival”, “survminer”, “limma”, and “ggpubr” R-packages were employed.

### Genomic Alterations PNPO in Pan-Cancer

The cBioPortal database (http://www.cbioportal.org/) was used to analyze the gene alternations of PNPO in TCGA pan-cancer datasets (Cerami et al., 2012). The genetic alterations and mutated site information of PNPO were obtained through the “Oncoprint”, “Cancer Type Summary”, and “Mutations” modules.

### Relationship Between PNPO Expression and Deoxyribonucleic Acid Mismatch Repair System, Microsatellite Instability, and Tumor Mutation Burden in Pan-Cancer

We performed the association analysis between PNPO expression and the related genes of MMR, MSI, and TMB due to increasing studies that showed that the prognosis of cancers was associated with them (Germano et al., 2017; Stelloo et al., 2017; Yu et al., 2019). Expression profile data from TCGA were used to evaluate the levels of the MMR genes. The result was visualized as a heatmap using the “reshape2” and “RColorBrewer” R-packages. The scores of MSI and TMB were used to be calculated from TCGA pan-cancer mutation data (https://tcga.xenahubs.net) (Bonneville et al., 2017). TMB scores were calculated by a Perl script and revised though dividing the total length of exons (Cheng et al., 2021). Spearman’s coefficient was performed to elaborate the correlations between the expression level of PNPO and MSI/TMB. Radar plots using the R-package “fmsb” were displayed as final results.

### Association Analysis of PNPO Expression with Tumor Immune Microenvironment in Cancers

The association between PNPO and immune checkpoint genes was processed by “reshape2” and “RColorBrewer” R-packages. The Estimation of Stromal and Immune cells in Malignant Tumors using Expression data (ESTIMATE) algorithm was performed to calculate immuneScore, stromalScore, and ESTIMATEScore by R package “ESTIMATE” (Yoshihara et al., 2013). The relationship between the expression of PNPO and the abundance of various immune-related cells in pan-cancer tissues was analyzed by the TIMER database (https://cistrome.shinyapps.io/timer/). PNPO expression in multiple types of cancer was evaluated through the “Diff Exp” module in the TIMER database (Li et al., 2017).

Biological Functions of PNPO by Gene Set Enrichment Analysis (GSEA)

Kyoto Encyclopedia of Genes and Genomes (KEGG) analysis was performed using the GSEA online database (https://www.gsea-msigdb.org/gsea/downloads.jsp). The biological functions of PNPO in pan-cancer were explored *via* GSEA analysis, which was processed with the R-packages “clusterProfiler” (Subramanian et al., 2005).

### Drug Sensitivity of PNPO in Pan-Cancer

NCI-60 compound activity data and RNA-seq expression profiles from the CallMiner™ were downloaded to analyze drug sensitivity of PNPO in pan-cancer (https://discover.nci.nih.gov/cellminer/home.do). Drugs approved by FDA or clinical trials were selected for analysis. The “impute”, “limma”, “ggplot2”, and “ggpubr” R package were performed (Shankavaram et al., 2009).

### Statistical Analysis

Wilcoxon rank-sum test and Spearman rank test were performed to examine the expression difference and correlation between two groups, respectively. Log-rank test was applied to analyze the survival outcome by Kaplan–Meier survival curves. Cox proportional hazard regression model was used to calculate the hazard ratio (HR). Statistical analyses were executed by GraphPad Prism8 and R version 4.0.2 software. Statistical significance was considered by *p*-values < 0.05 (*p < 0.05, **p < 0.01, ***p < 0.001, and ****p < 0.0001).

## Results

### Expression of PNPO in Pan-Cancer and Normal Tissues

**FIGURE 1:**
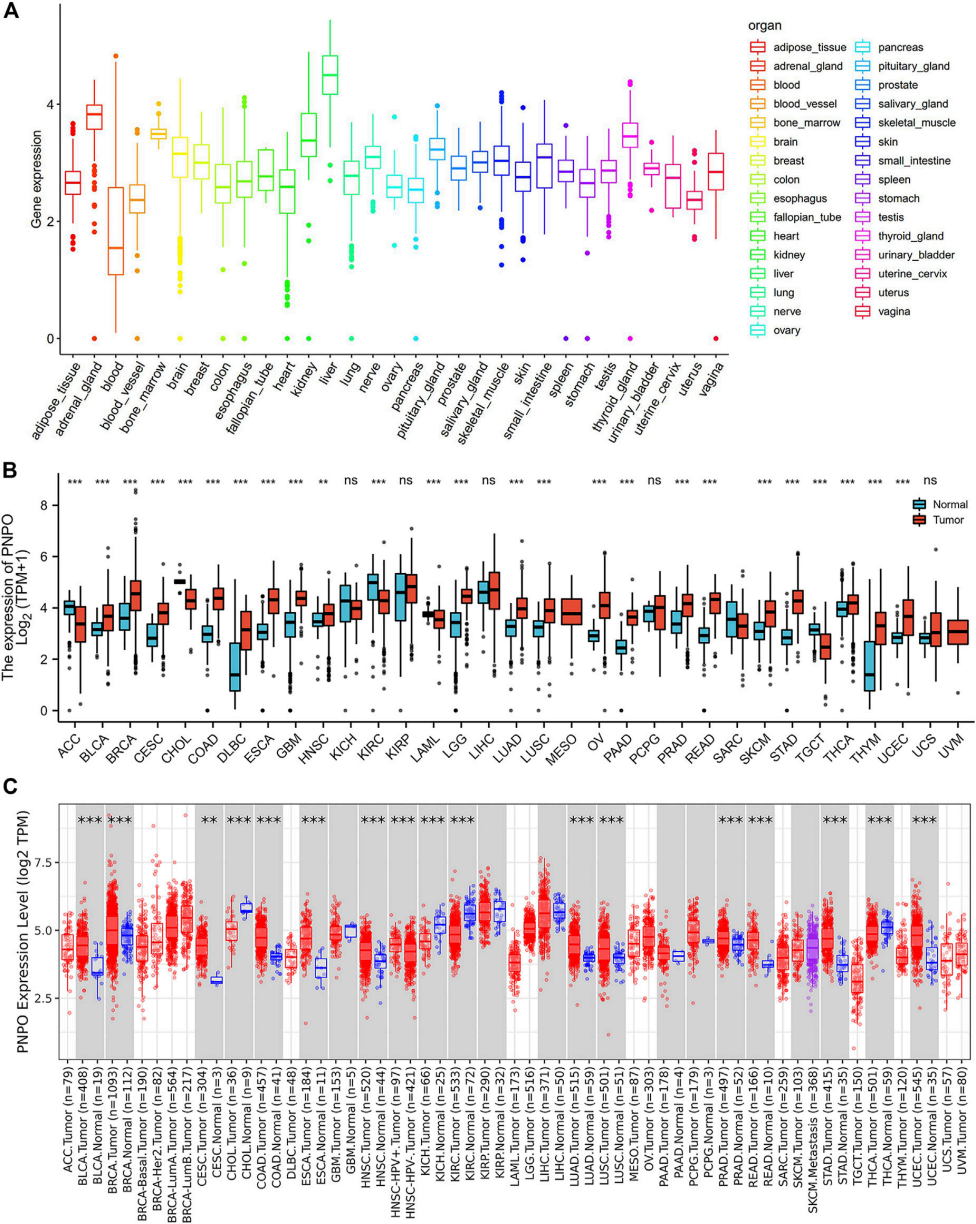
Differential expression of PNPO. **(A)** PNPO mRNA expression in normal tissues from GTEX data. **(B)** Differential PNPO mRNA expression between TCGA cancers and GTEX normal tissues. The red column represents cancer samples and the blue column represents normal samples. Normal group was normal tissue in TCGA and GTEX database. **(C)** PNPO mRNA expression in different cancer types in TIMER. *p < 0.05, **p < 0.01, and ***p < 0.001. Normal group was normal tissue in TCGA database.

To explore the mRNA expression of PNPO in human normal tissues, we studied PNPO expression in physiological tissue from the GTEX dataset. PNPO was overexpressed in the liver, adrenal gland, and kidney tissues (Figure 1A). The expression abundances of PNPO in various male and female tissues were also shown. In sum, there was no significant difference between PNPO mRNA expression levels and gender (Supplementary Figure S1A). Next, the expression of PNPO in pan-cancer was further analyzed through the RNA-seq data of TCGA and GTex databases. A significant expression difference of PNPO was found in 33 types of cancer excluding those without normal tissue data. PNPO expression was overexpressed in BLCA (bladder urothelial carcinoma), BRCA (breast invasive carcinoma), CESC (cervical squamous cell carcinoma and endocervical adenocarcinoma), CHOL (cholangiocarcinoma), COAD (colon adenocarcinoma), DLBC (lymphoid neoplasm diffuse large B-cell lymphoma), ESCA (esophageal carcinoma), GBM (glioblastoma multiforme), HNSC (head and neck squamous cell carcinoma), LGG (brain lower grade glioma), LUAD (lung adenocarcinoma), LUSC (lung squamous cell carcinoma), OV (ovarian serous cystadenocarcinoma), PAAD (pancreatic adenocarcinoma), PRAD (prostate adenocarcinoma), READ (rectum adenocarcinoma), SKCM (skin cutaneous melanoma), STAD (stomach adenocarcinoma), THCA (thyroid carcinoma), THYM (thymoma), and UCEC (uterine corpus endometrial carcinoma) compared to control tissues (Figure 1B). On the contrary, PNPO was decreased in ACC (adrenocortical carcinoma), KIRC (kidney renal clear cell carcinoma), LAML (acute myeloid leukemia), and TGCT (testicular germ cell tumors) compared to control tissues (Figure 1B). We also searched the mRNA expression of PNPO in human pan-cancer by the TIMER database. PNPO expression was significantly upregulated in BLCA, BRCA, CESC, COAD, ESCA, GBM, HNSC, LUAD, LUSC, PAAD, PCPG (pheochromocytoma and paraganglioma), PRAD, READ, STAD, and UCEC (Figure 1C), while PNPO was downregulated in CHOL, KICH (kidney chromophobe), KIRC, and THCA (Figure 1C) than the control group. Based on the Oncomine database, PNPO expression was higher expressed in colorectal cancer, leukemia, lymphoma, and myeloma compared to the human normal control tissues, while it was lower expressed in kidney cancer (Supplementary Figure S1B). PNPO was overexpressed in almost all the human cell lines in the CCLE database except the medulloblastoma cell line (Supplementary Figure S1C).

### Protein Expression of the PNPO in Human Tissues

**FIGURE 2:**
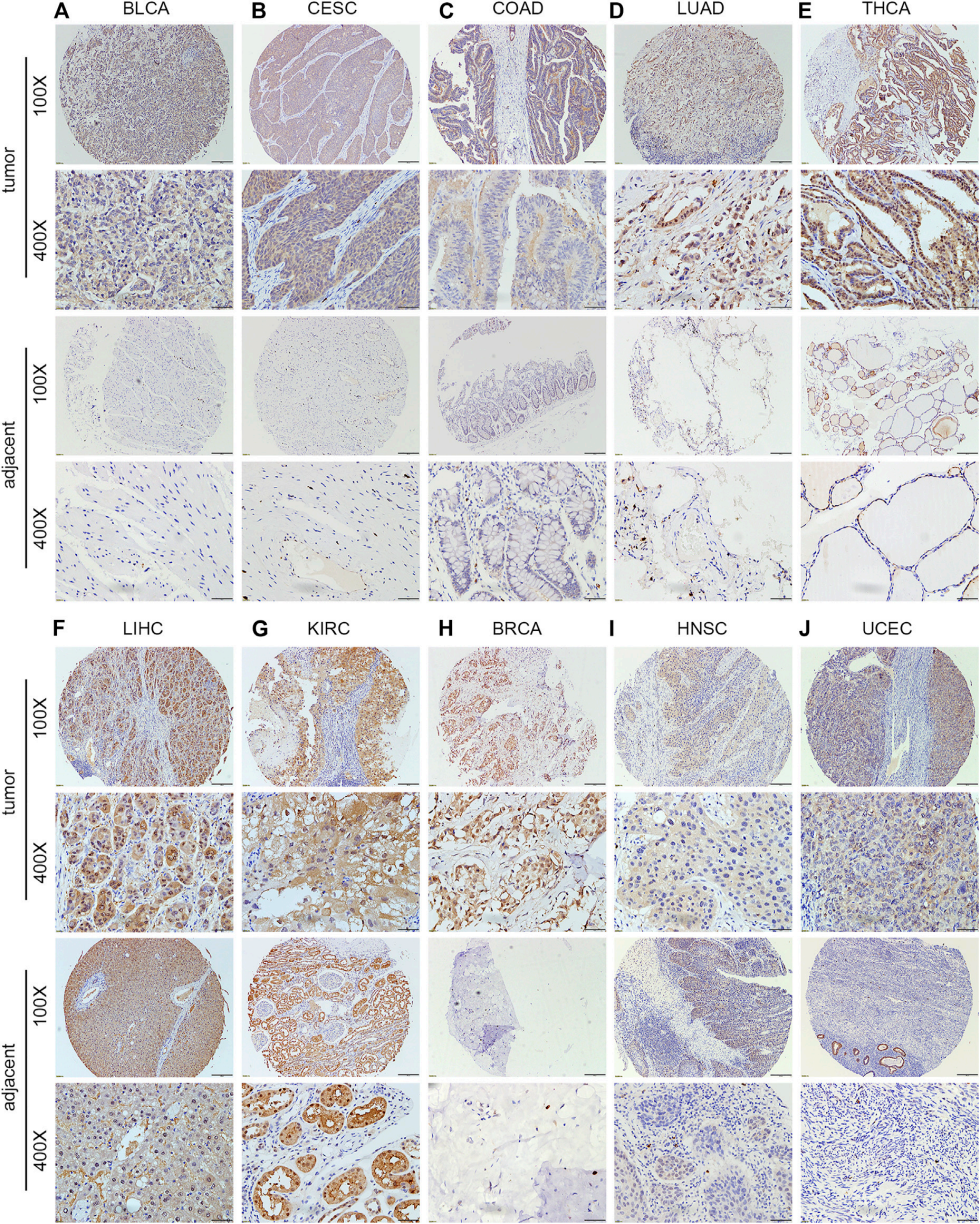
Protein expression level of PNPO in human multiple cancer tissues of BLCA **(A)**, CESC **(B)**, COAD **(C)**, LUAD **(D)**, THCA **(E)**, LIHC **(F)**, KIRC **(G)**, ESCA **(H)**, LUSC **(I)**, and TGCT **(J)**. Representative images of PNPO expression in pan-cancer tissues are shown. Original magnification, ×100 and ×400.

The human multiple cancer tissue microarray data showed that PNPO expression was negative in the normal esophagus (*n* = 2), rectum (*n* = 2), thyroid (*n* = 4), and lung (*n* = 4) tissues, while it was positive in the normal stomach (*n* = 2), colon (*n* = 2), liver (*n* = 1), and pancreas (*n* = 1) tissues (Supplementary Figure S2). The staining intensity of PNPO was greater in BLCA (*n* = 16), CESA (*n* = 8), COAD (*n* = 5), LUAD (*n* = 5), and THCA (*n* = 12) than their adjacent tissues (*p* < 0.05, Figures 2A–E and Supplementary Table S1). The protein expression level of PNPO was strongly expressed in both normal and cancer tissues of the liver (*n* = 5) and kidney (*n* = 5) (Figures 2F,G). PNPO was also expressed in a major percentage of certain cancer types, such as BRCA (*n* = 7), HNSC (*n* = 3), and UCEC (*n* = 8) without significant difference (Figures 2H–J). From the results, we deduced that PNPO might be positively expressed in these cancers if we enlarge the sample size. The protein expression level of PNPO was low in LUSC (*n* = 4), TGCT (*n* = 3), PESC (penis squamous cell carcinoma, *n* = 5), ESCA (*n* = 5), STAD (*n* = 5), and READ (*n* = 3), which also has no significant difference (Supplementary Figures S3A–F). We speculate that there are two possible reasons for this situation: the sample size is not enough and the heterogeneity of the tumor (Dagogo-Jack and Shaw, 2018; Lawson et al., 2018). PNPO protein was expressed in pancreas normal and adjacent tissues, while not expressed in pancreas cancer (Supplementary Figure S3G), which was not consistent with its mRNA expression level. Our previous data showed that PNPO was negative in normal ovarian and fallopian tube tissues. Despite the lack of negative control tissue of the ovary in this array, we found that PNPO staining was strong in ovarian cancer tissues (*n* = 6) (Supplementary Figure S3H). The staining of PNPO was also strong in prostate cancer (*n* = 6) and brain tumor (*n* = 6) tissues, but not in their adjunct tissues or normal tissues in this tissue array (Supplementary Figures S3I,J).

### Prognostic Assessment Value of PNPO in Pan-Cancer

**FIGURE 3:**
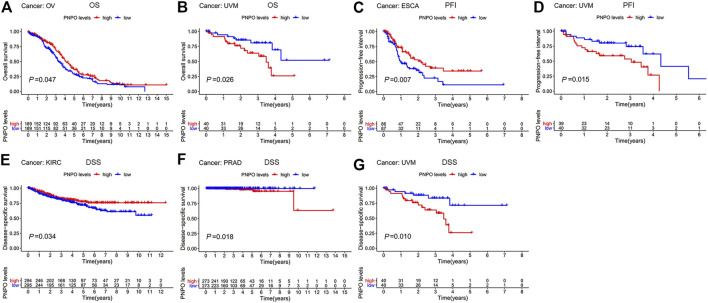
Patient survival period analysis. **(A,B)** Kaplan–Meier analysis of the association between PNPO expression and OS in OV **(A)**, and UVM **(B)**. **(C,D)** Kaplan–Meier analysis of the correlation between PNPO expression and PFI in ESCA **(C)**, and UVM **(D)**. **(E,G)** Kaplan–Meier analysis of the association between PNPO expression and DSS in KIRC **(E)**, PRAD **(F)**, and UVM **(G)**. The red line represents high PNPO expression and the blue line represents low PNPO expression. OS, overall survival; PFI, progression-free interval; DSS, disease-specific survival.

In order to survey the prognostic assessment value of PNPO in pan-cancer, Cox proportional hazards model and Kaplan–Meier analysis were carried out to evaluate the relationship between PNPO expression and patients’ survival period. The expression of PNPO was correlated with OS in KIRC (*p* = 0.003), LAML (*p* = 0.014), and THYM (*p* = 0.041) performed by Cox proportional hazards regression model. PNPO was regarded as a high-risk factor for LAML and THYM, while it was a low-risk factor for KIRC of OS prognosis assessment in pan-cancer (Supplementary Table S2). A high level of PNPO predicted good OS in OV (*p* = 0.047, Figure 3A), while a high level of PNPO predicted poor OS of UVM (uveal melanoma) by Kaplan–Meier survival analysis (*p* = 0.0261, Figure 3B). For PFI, the overexpressed mRNA level of PNPO represented an adverse factor in BLCA (*p* = 0.038), while the overexpressed PNPO was a favorable factor in KIRC (*p* = 0.004) (Supplementary Table S2). Kaplan–Meier curves for PFI indicated a positive correlation between PNPO overexpression and good survival outcome in patients with ESCA (*p* = 0.007, Figure 3C) but a negative relationship between PNPO expression and PFI in UVM (*p* = 0.015, Figure 3D). Next, we found that high PNPO expression predicted poor DFI in BLCA (*p* = 0.034) (Supplementary Table S2), but there was no significance in pan-cancer by Kaplan–Meier survival analysis. Furthermore, PNPO exhibited a significant prognostic value in KIRC (*p* < 0.001), PRAD (*p* = 0.002), and UVM (*p* = 0.034) in Cox proportional hazards regression model for DSS (Supplementary Table S2). Patients with the overexpression of PNPO had lengthened DSS in KIRC (*p* = 0.034, Figure 3E), but had shortened DSS in PRAD (*p* = 0.018, Figure 3F) and UVM (*p* = 0.010, Figure 3G).

### Correlation Analysis Between PNPO Expression and Clinicopathological Phenotypes in Pan-Cancer

We further explored the correlation between the mRNA expression level of PNPO and patients’ clinicopathological features progression in pan-cancer. Higher expression of PNPO was found in age ≥65 years group in BRCA (*p* = 0.0044), KICH (*p* = 0.033), LIHC (*p* = 0.01), PCPG (*p* = 0.0089), and THYM (*p* = 0.036, Supplementary Figure S4A). PNPO expression was related to tumor stage in LUSC, PAAD, READ, STAD, and THCA (Supplementary Figure S4B). We also discovered that PNPO was associated with tumor treatment response in BLCA, DLBC, KIRC, PRAD, and UCEC, especially between CR and PR groups (Supplementary Figure S4C). Then, we found that the overexpression of PNPO was significantly associated with tumor status in BLCA and GBM, while the overexpression of PNPO was correlated with tumor-free status in KIRC and SARC (sarcoma) (Supplementary Figure S4D). Moreover, we investigated the relationship between the mRNA expression of PNPO and clinical characteristics of BRCA patients through the Kaplan–Meier plotter database (Supplementary Table S3). Overexpressed PNPO was associated with better OS and PFS in ER-positive, HER2-negative, and Grade 3 BRCA patients (*p* < 0.05). High PNPO mRNA expression level was associated with better OS in the lymph node-positive and -negative groups, while it was only correlated with better RFS in the lymph node-positive group. The overexpression of PNPO was also positively related to better RFS in the HER2-positive group patients. Meanwhile, overexpressed PNPO was also associated with better RFS during luminal A and B groups. These results suggest that PNPO expression level could impact the prognosis in BRCA patients.

### Genetic Alteration Analysis of PNPO in Pan-Cancer

**FIGURE 4:**
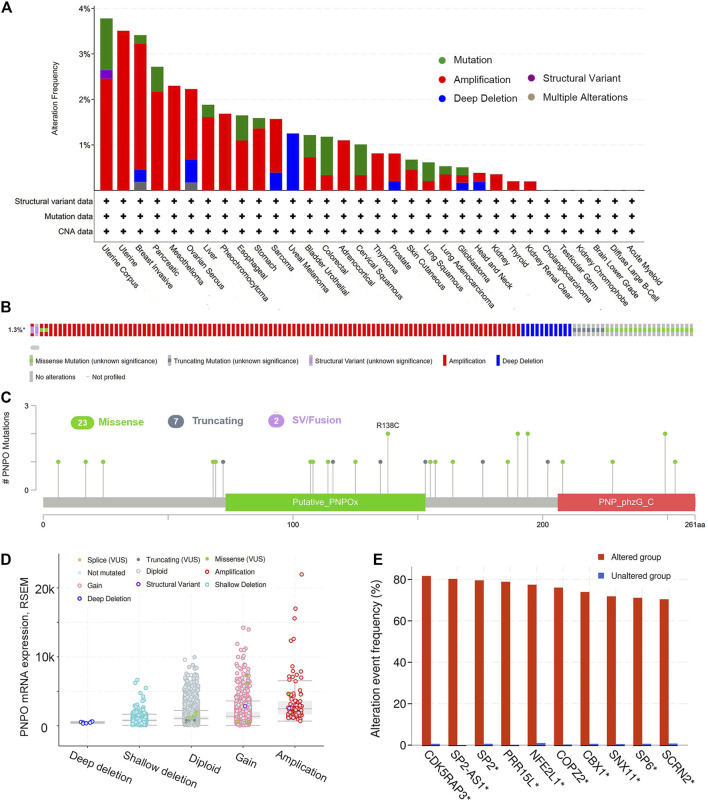
The genetic alterations of PNPO. **(A)** Alterations summary of PNPO in TCGA pan-cancer datasets. **(B)** Summary of PNPO structural variant, mutations, and copy-number alterations. **(C)** The mutation types, number, and sites of the PNPO genetic alterations. **(D)** The alteration types of PNPO in pan-cancer. **(E)** The related genes alteration frequency in PNPO altered group and unaltered group.

The highest gene alteration rate of PNPO appears for pan-cancer patients with uterine corpus endometrial carcinoma, uterine carcinosarcoma, breast invasive carcinoma, pancreatic adenocarcinoma, and mesothelioma with amplification (>2%) as the primary type using the cBioPortal database (Figure 4A). Amplification, miss mutation, and deep deletion are the main type of frequent genetic alterations of PNPO (Figure 4B). The types, sites, and case numbers of the *PNPO* gene modification were further displayed (Figure 4C). *PNPO* missense mutation was the main type alteration, while R138C alteration was detected in 2 cases of UCEC. The most frequent putative copy-number alterations of PNPO were amplification, gain function, and diploid (Figure 4D). The gene alteration of CDK5RAP3, SP2-AS1, SP2, PRR15L, NFE2L1, COPZ2, CBX1, SNX11, SP6, and SCRN2 was more common in the altered group than in the unaltered group (Figure 4E).

### Correlation of PNPO Expression With Immune-Related Biomarker

**FIGURE 5:**
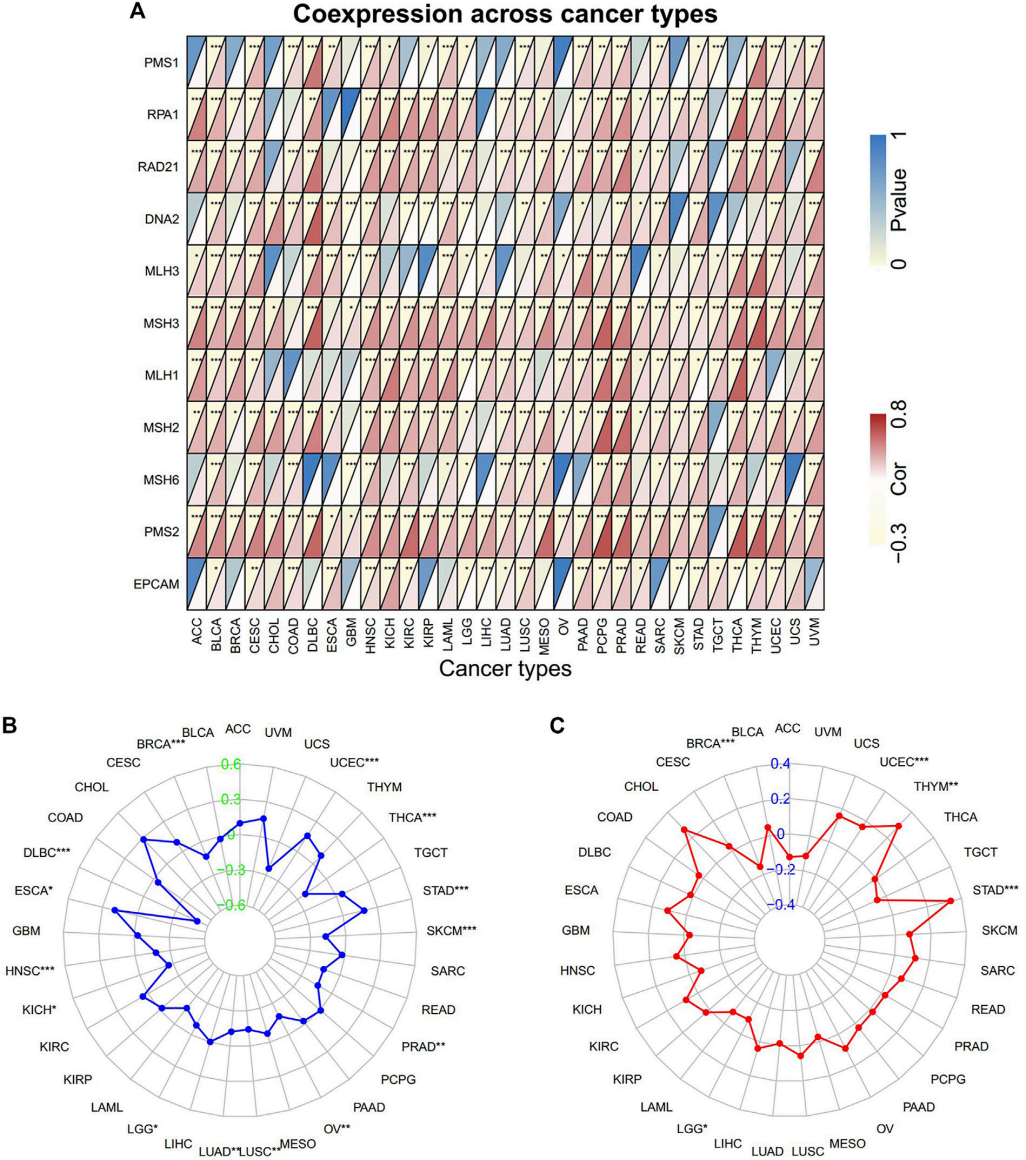
The correlation of PNPO expression with immune-related biomarkers. **(A)** Heatmap indicating the correlation between PNPO expression and mismatch repair (MMR) genes. For each pair, the top left triangle indicates the *p*-value, and the bottom right triangle indicates the correlation coefficient. **(B)** Radar map of correlation between PNPO expression and microsatellite instability (MSI). **(C)** Radar map of correlation between PNPO expression and tumor mutation burden (TMB). *p < 0.05, **p < 0.01, and ***p < 0.001.

The correlation between PNPO expression and MMR/MSI/TMB across all tumors of TCGA was further analyzed. PNPO expression was positively correlated with MMR-related genes level in most tumors, especially in BLCA, CESC, HNSC, LUSC, PRAD, and STAD (Figure 5A). In addition, the expression of PNPO also has a positive correlation with the most common MMR-related genes, including MLH1, PMS2, MSH2, and MSH6 in KIRC, LAML, LGG, LUAD, PCPG, SARC, SKCM, YHCA, and UVM. The results displayed that PNPO expression was positively associated with increased MSI in ESCA, STAD, and UCEC, while PNPO expression was negatively associated with MSI in BRCA, DLBC, HNSC, KICH, LGG, LUAD, LUSC, OV, PRAD, SKCM, and THCA (Figure 5B). Additionally, a positive association between PNPO expression and TMB was displayed in STAD, THYM, and UCEC, while a negative association between PNPO mRNA expression and TMB was found in BRCA and LGG (Figure 5C).

### Correlation of PNPO Expression With Tumor Immune Microenvironment

**FIGURE 6:**
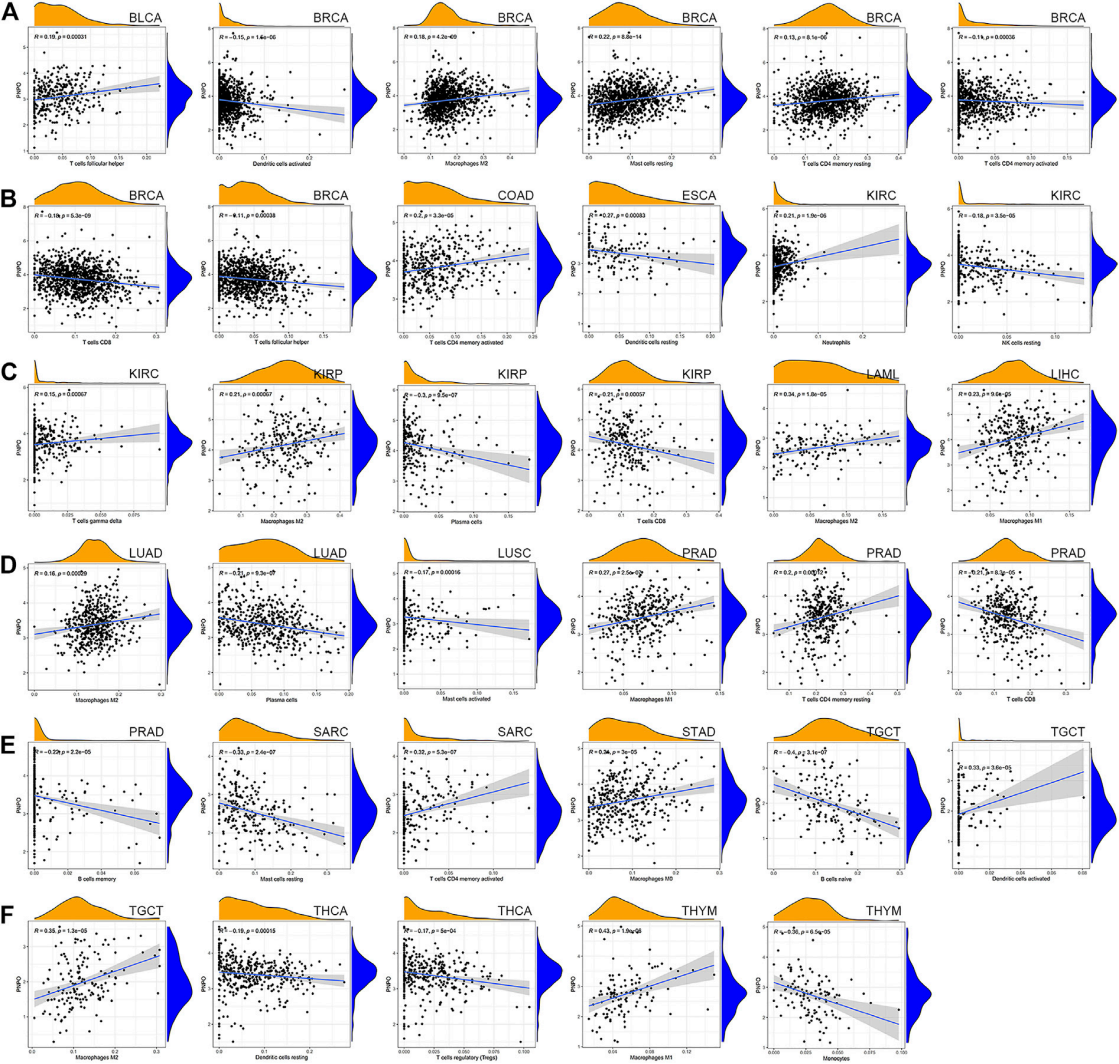
Correlation between PNPO gene expression and tumor immune microenvironment in TCGA database. **(A–F)** Analysis of immune-associated cells infiltration with PNPO expression in pan-cancer.

To further investigate the relationship with the human immune system, we examined the relationship between PNPO expression and the tumor immune microenvironment according to the ESTIMATE algorithm and TIMER database. It was shown that PNPO expression had a positive correlation with estimated immune and stromal scores in GBM, while a negative correlation with immune and stromal scores was found in BRCA, PAAD, PCPG, STAD, THCA, and UCEC (Supplementary Table S4). The association between the expression of PNPO and immune checkpoint genes expression was explored. The results demonstrated that the mRNA expression level of PNPO was significantly correlated with mostly immune checkpoint genes, which suggested that a high level of PNPO might mediate immune escape. In particular, we found that in BRCA, HNSC, LIHC, SARC, THCA, and UVM, PNPO expression was correlated with more than 30 immune checkpoint markers, such as TNFRSF, CD48, and ICOS (Supplementary Figure S5). The relationship between PNPO expression and immune-associated cells infiltration in pan-cancer was further performed in the TIMER database. It was shown that PNPO expression was significantly correlated with six types of infiltrating immune-associated cells including B cell, CD8+T cells, CD4+T cells, neutrophils, macrophages, and dendritic cells in BRCA, GBM, KICH, LIHC, SARC, STAD, THCA, and THYM (Supplementary Figure S6). In particular, we found that the mRNA expression level of PNPO was correlated with 8 types of immune-associated cells in BRCA (Figures 6A,B). Furthermore, we also found that the expression of PNPO was significantly correlated with the immune-associated cell infiltration levels of macrophages in 9 cancer types, CD4+T cells in 4 cancer types, dendritic cells in 3 cancer types, mast cells in 3 cancer types, and CD8+T cells in 3 cancer types in the ESTIMATE algorithm (Figure 6).

### Biological Function of PNPO in Cancer

**FIGURE 7:**
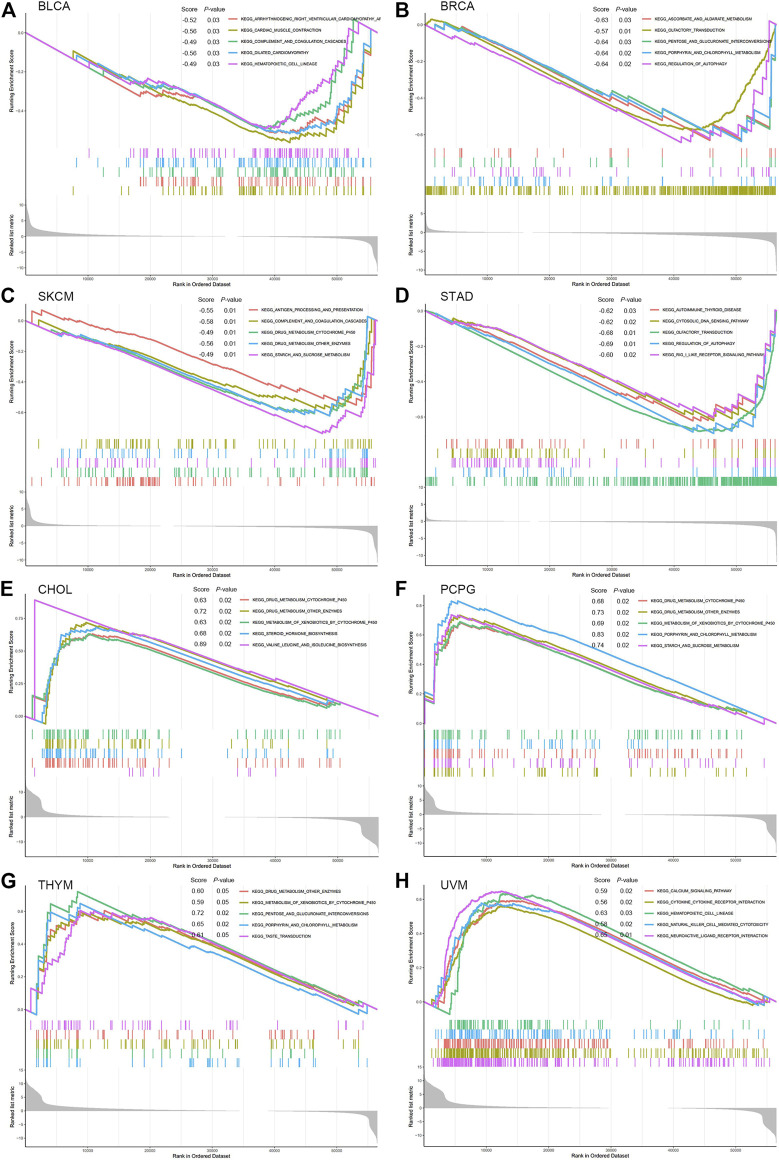
GSEA analysis in KEGG signature of PNPO in BLCA **(A)**, BRCA **(B)**, SKCM **(C)**, STAD **(D)**, CHOL **(E)**, PCPG **(F),** THYM **(G)**, and UVM **(H)**. Different color curves represent different functions or pathways. The peak of the upward and downward curve indicates the positive and negative regulation of PNPO, respectively. Score, enrichment score.

GSEA was performed to explore the main biological process affected by PNPO in pan-cancer. From KEGG gene sets analysis, the data suggested that PNPO negatively regulated signaling pathways in BLCA, BRCA, SKCM, and STAD, while PNPO positively regulated signaling pathways in CHOL, PCPG, THYM, and UVM (Figure 7). We also found that PNPO had a complex regulation with different signaling pathways in MESO (mesothelioma), OV, SARC, and UCEC (Supplementary Figure S7). Drug metabolism was the most common signaling pathway of PNPO involved in pan-cancer, followed by hematopoietic cell lineage, porphyrin, and chlorophyll signaling pathway. Antigen processing and presentation, natural killer cell-mediated cytotoxicity, cytokine–cytokine receptor interaction, and regulation of autophagy were all involved in PNPO biology function in pan-cancer biological analysis.

### Drug Sensitivity Analysis of PNPO

**FIGURE 8:**
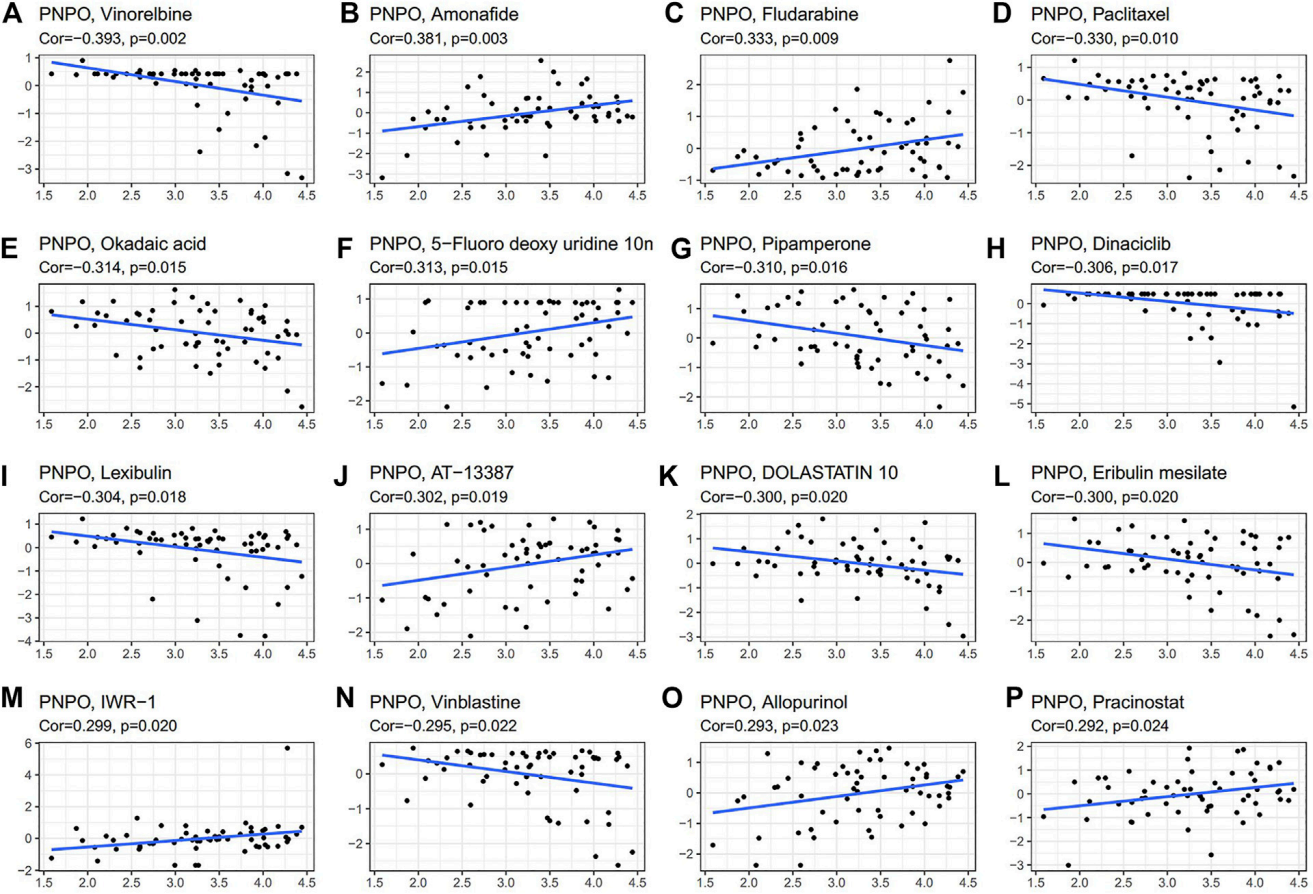
Drug sensitivity analysis of PNPO. The expression of PNPO was associated with the sensitivity of vinorelbine **(A)**, amonafide **(B)**, fludarabine **(C)**, paclitaxel **(D)**, okadaic **(E)**, 5-fluoro deoxyuridine **(F)**, pipamerone **(G)**, dinaciclib **(H)**, lexibulin **(I)**, AT-13387 **(J)**, dolastatin **(K)**, eribulin mesilate **(L)**, IWR-1 **(M)**, vinblastine (**N)**, allopurinol **(O)**, and pracinostat sensitivity (**P)**.

We further investigated the potential correlation analysis between drug sensitivity and PNPO expression using the CellMiner™ database. Notably, PNPO expression was negatively correlated with drug sensitivity of vinorelbine, paclitaxel, okadaic acid, pipamerone, dinaciclib, lexibulin, dolastatin, eribulin mesilate, and vinblastine (Figures 8A,D,E,G–I,K,L,N). Our results exhibited that PNPO expression was positively associated with amonafide, fludarabine, 5-fluoro deoxyuridine, AT-13387, IWR-1, allopurinol, and pracinostat sensitivity (Figures 8B,C,F,J,M,O,P). The data indicated that PNPO might be associated with chemoresistance of certain chemotherapeutic agents, such as paclitaxel and vinblastine, which were commonly used in the clinic. Comprehensively, we deduced that the involvement of PNPO in chemoresistance might be related to the metabolism of starch and sucrose, porphyrin and chlorophyll, cytochrome P450, and other enzymes, and the biosynthesis of isoleucine and other amino acids (Figures 7, 8).

## Discussion

PNPO is an enzyme that converts pyridoxine 5′-phosphate into PLP, an active form of vitamin B6 implicated in a variety of cancers (Musayev et al., 2009). PNPO, which served as a key enzyme in the metabolism of vitamin B6, has primarily been studied in the context during the development of epilepsy (Hoffmann et al., 2007; Bagci et al., 2008). The deficiency of PNPO was verified to be a reason for infantile and neonatal epileptic encephalopathy (Khayat et al., 2008). PNPO deficiency could also lead to hepatic cirrhosis, which may be associated with apparent epigenetic activation of purinergic signaling in hepatic stellate cells (Coman et al., 2016). Our previous studies confirmed that PNPO was overexpressed in ovarian cancer and enhanced the malignant biology function of ovarian cancer cells, indicating that PNPO is a candidate oncogene role in tumorigenesis (Zhang et al., 2017). However, the research of PNPO in other kinds of cancer types is still very poor. Thus, we produced the research focus on the roles of PNPO in human pan-cancer in this study. The mRNA expression level of PNPO is relatively overexpressed in the human liver, kidney, and skeletal muscle, but low expressed in the ovary and lung (Kang et al., 2004). The results were verified according to the GTEX dataset, indicating that PNPO was overexpressed in the liver, adrenal gland, and kidney tissues. This is similar to our finding that PNPO was highly expressed in stomach, colon, liver, and pancreas normal tissues in protein expression level by immunohistochemistry. We next confirmed the expression of PNPO in 33 tumor types compared to normal tissues from the online database. PNPO was significantly upregulated in most types of cancer, while it was only found to be downregulated in ACC, KIRC, LAML, and TCGT compared to control tissues. Based on the CCLE database, we discovered that PNPO was overexpressed in most cell lines. By our multiple organ tissue arrays, the protein expression of PNPO was higher in BLCA, CESC, COAD, LUAD, and THCA tissues. The previous studies showed that PNPO was upregulated in ovarian cancer (Zhang et al., 2017) and human breast invasive ductal carcinoma patients (Ren et al., 2019). PNPO was also reported to be upregulated in colorectal cancer from a gene array study (Chen et al., 2017). In our previous study, it was unveiled that increased PLP could inhibit the protein expression of PNPO, thus suppressing the proliferation of human ovarian cancer cells (Zhang et al., 2017). High circulating levels of PLP, reflecting reduced amounts of circulating homocysteine, have been associated with improved disease outcomes in pan-cancer patients (Galluzzi et al., 2013). Elevated PLP was assumed or verified to decrease cell proliferation in melanoma, hepatoma, gastric cancer, breast cancer, and pancreatic cancer cell lines (Peterson et al., 2020). These reports were consistent with our results and further confirmed with our data, suggesting that PNPO took important effects on tumorigenesis during human cancers.

Furthermore, we try to analyze the correlation between the mRNA expression level of PNPO and the prognosis in human pan-cancer patients. Our Kaplan–Meier survival indicated that PNPO was negatively related with the OS in BLCA, PFI in UVM, DSS in PRAD, and UVM with a significant difference. These could confirm the oncogenic role of PNPO in human cancers. However, we also found that the mRNA expression level of PNPO acted as a protective factor in OV and KIRC. These suggested that PNPO may play different roles in certain cancer types, which need further to be tested in the future. Additionally, it has been shown that high expression of PNPO was associated with clinical stages and metastasis in breast invasive ductal carcinoma (Ren et al., 2019), but we found that PNPO was more likely a protective factor in BRCA patients from Kaplan–Meier plotter databases. We suspected that the difference between our results from the online database and the reported data might be due to the different methods to test the expression of PNPO. The protein expression of PNPO in human breast invasive ductal carcinoma was examined (Ren et al., 2019), while mRNA expression of PNPO in BRCA was tested in Kaplan–Meier plotter databases.

The *PNPO* gene is located in chromosome 17q21.32 (Mills et al., 2014). PNPO deficiency, due to its gene mutations, has been widely studied in infantile and neonatal epileptic encephalopathy (Mills et al., 2005; Hoffmann et al., 2007; Hatch et al., 2016; di Salvo et al., 2017; Ciapaite et al., 2020). However, there is no related research about PNPO gene alteration in human cancers. We then unearthed the fact that amplification was the most altered frequency of PNPO in pan-cancer using the cBioPortal database. It was found that the co-occurrence of CDK5RAP3, SP2-AS1, SP2, PRR15L, NFE2L1, COPZ2, CBX1, SNX11, SP6, and SCRN2 alterations was observed with the PNPO alteration group. It was suggested that PNPO played an important role in the maintenance of DNA integrity and glucose homeostasis in *Drosophila* (Mascolo et al., 2020). The inhibitive effect of PNPO on the glycine cleavage system could cause synthetic lethality in *Escherichia coli* (Ito et al., 2020). PNPO is a converting enzyme for PLP, which acts as a co-factor for more than 140 enzymes in metabolism. PLP, which served as an active form of vitamin B6, took part in many metabolic processes such as one-carbon, amino acid, gluconeogenesis, and lipid, contributing to tumorigenesis (Brasky et al., 2017). The amplification of PNPO in pan-cancer may induce the abnormal expression of PLP (Wilson et al., 2019), which leads to the dysfunction of physical metabolism in cancer development.

In the study, we first displayed evidence of the potential association between the expression of PNPO and MMR, MSI, or TMB. The results revealed that PNPO expression was positively related to MMR-related gene expression in most types of tumors. Additionally, PNPO correlates with MLH1, MSH2, MSH6, and PMS2 in KIRC, LAML, LGG, LUAD, PCPG, SARC, SKCM, YHCA, and UVM. Further analysis demonstrated that the expression of PNPO is significantly associated with MSI in 14 cancer types and TMB in 4 cancer types. The data indicated that PNPO expression might influence the response of cancer patients to immune checkpoint therapy, which will contribute to further understand the mechanism of immunotherapy in treating cancers.

A previous study suggested that a lack of vitamin B6 may damage immune response (Doke et al., 1997). Low plasma PLP is related to impaired differentiation and maturation of monocyte-derived macrophages and T lymphocytes (Meydani et al., 1991; Rall and Meydani, 1993). Vitamin B6 deficiency significantly reduced the percentage and total number of lymphocytes, mitogenic responses of peripheral blood lymphocytes to mitogens, and the production of interleukin 2 (Meydani et al., 1991). PLP was strongly negatively correlated with tumor volume and increased lymphocyte proliferation in mice, indicating that PLP may have a greater antitumor immune response (Gebhard et al., 1990). This indicated that PLP, the active form of Vitamin B6, played an important role in regulating the human immune system. Therefore, we suspected that PNPO as the key enzyme-producing PLP could also take part in immunity regulation in human cancer. Our correlation analysis demonstrated that more than 30 immune checkpoint genes were positively correlated with PNPO expression in many tumor types, including BRCA, HNSC, LIHC, SARC, THCA, and UVM. The results suggested that PNPO might be involved in immune escape in human cancer immune therapy. TIMER database mining further found that PNPO expression was significantly associated with the infiltration levels of various immune-associated cells, including B cell, dendritic cells, CD8+T cells, CD4+T cells, neutrophils, and macrophages in BRCA, GBM, KICH, LIHC, SARC, STAD, THCA, and THYM. According to the ESTIMATE algorithm, the correlation between PNPO expression and immune-associated cell infiltration also occurred in many types of cancer, including BRCA, KIRC, KIRP, and PRAD. Until now, little is known about the roles that PNPO plays in the human immune system. The roles of PNPO in the tumor immune microenvironment remains a research gap worth investigating in further research.

From KEGG analysis, we found that drug metabolism is the most common signaling pathway of PNPO involved in pan-cancer. PNPO is the key enzyme to produce PLP, which has been estimated to be a co-factor in about 4% of human enzyme activities (Ueland et al., 2017). It is no surprise that PNPO has been associated with human physical metabolism. However, there is no related research between PNPO and drug sensitivity or resistance until now. Using the CellMiner™ database, we found that PNPO expression was correlated with many drug sensitivity, such as paclitaxel, vinblastine, and 5-fluoro deoxyuridine. Therefore, we deduced that PNPO may take effect in chemotherapy and may be correlated with chemoresistance. Antigen processing and presentation, and natural killer cell-mediated cytotoxicity were identified gene regulatory mechanisms of PNPO from KEGG analysis, which suggested these were the candidate signaling pathways associated with PNPO and immune system in pan-cancer. We found that TGF-β1 signaling-mediated PNPO expression was at least partially mediated by the upregulation of miR-143–3p in ovarian cancer (Zhang et al., 2017). PNPO could be regulated by the MALAT1/miR-216b-5p/PNPO axis in the development of human breast invasive ductal carcinoma (Ren et al., 2019). Thus, these data implicated that PNPO may crosstalk between vitamin B6 mechanism and other signaling pathways. We speculate that the mechanism of PNPO involved in human cancers could include two parts: one is involved in metabolic regulation, and the other is to function through interaction with signaling pathways. The regulation of metabolism may be the common thing with PNPO in various tumors, while participating in the regulation of different signal pathways may be the reason for its heterogeneity in pan-cancer.

## Conclusion

The pan-cancer analysis systematically displayed the characteristics of PNPO in multiple aspects, including expression pattern, survival prognosis, genetic mutation, MMR, MSI, TMB, tumor immune microenvironment, signaling pathway, and drug sensitivity. PNPO might serve as a potential target for cancer treatment since they displayed abnormal expression in multiple cancers and predicted a worse prognosis in cancer patients. Moreover, the aberrant PNPO expression was related to MMR, MSI, TMB, and tumor immune microenvironment across various types of cancer. This study highlights the multifaceted roles of PNPO in pan-cancer and provides new insights into the candidate effect of PNPO in regulating chemoresistance.

## Data Availability

The original contributions presented in the study are included in the article/Supplementary Material. Further inquiries can be directed to the corresponding authors. Tissue microarray data was deposited at 10.5061/dryad.rfj6q57c3
