# Supplementary figures and images for: Prognostic Implication and Oncogenic Role of PNPO in Pan-Cancer
Source: Front Cell Dev Biol. 2022 Jan 21;9:763674. doi: 10.3389/fcell.2021.763674 (PMC8814662; doi:10.3389/fcell.2021.763674)

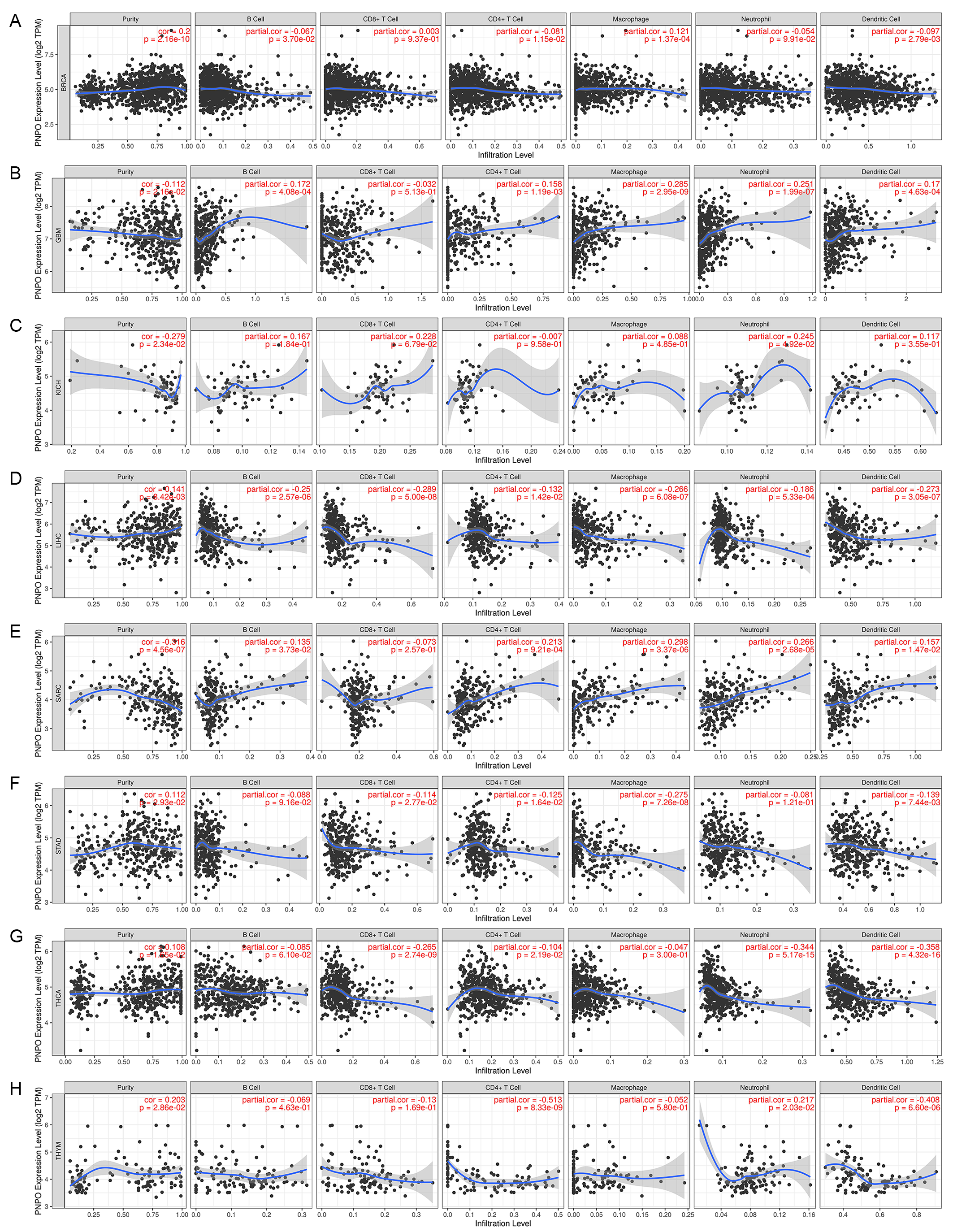

Supplement: Supplementary file 1 [file Image6.TIF]

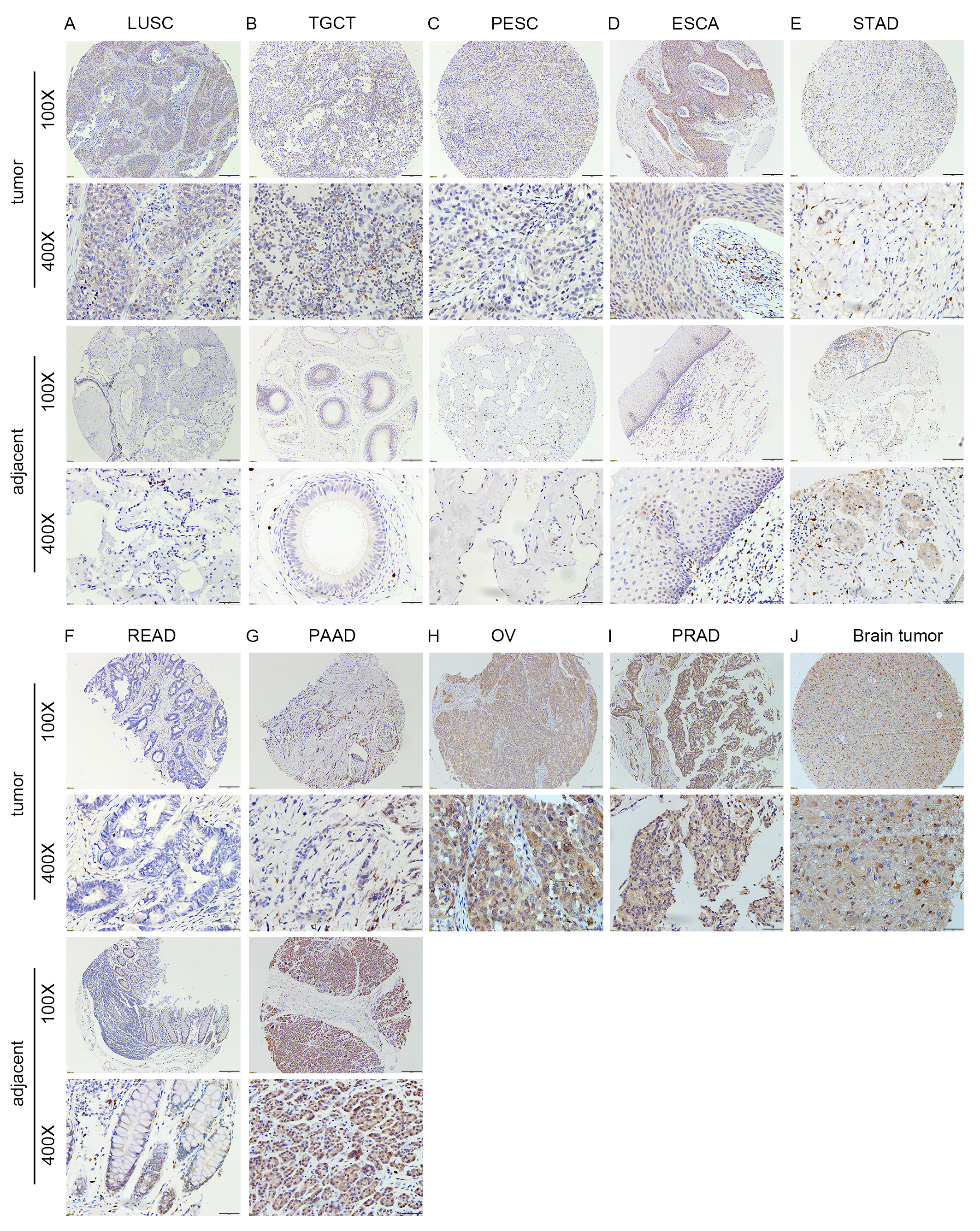

Supplement: Supplementary file 3 [file Image3.TIF]

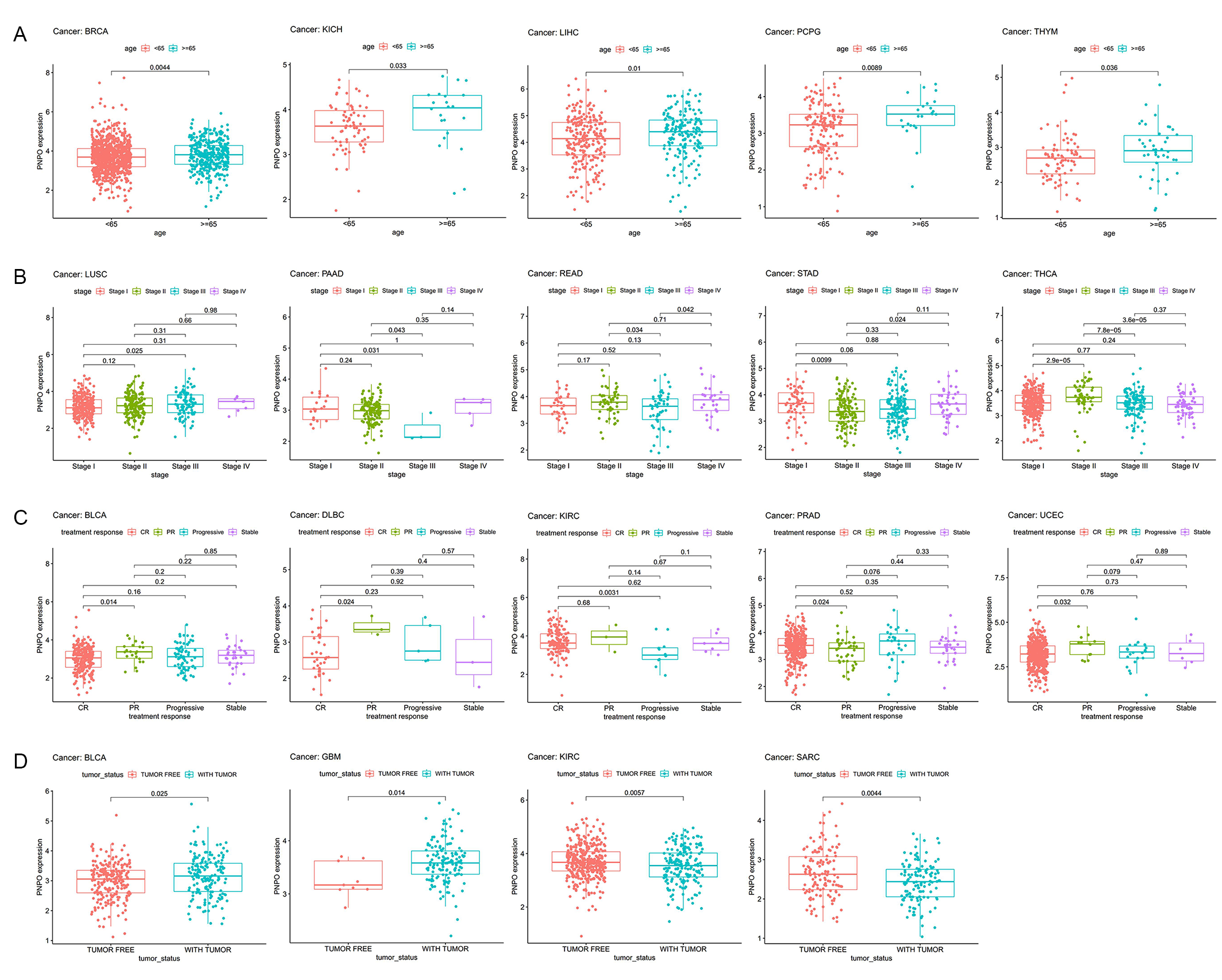

Supplement: Supplementary file 4 [file Image4.TIF]

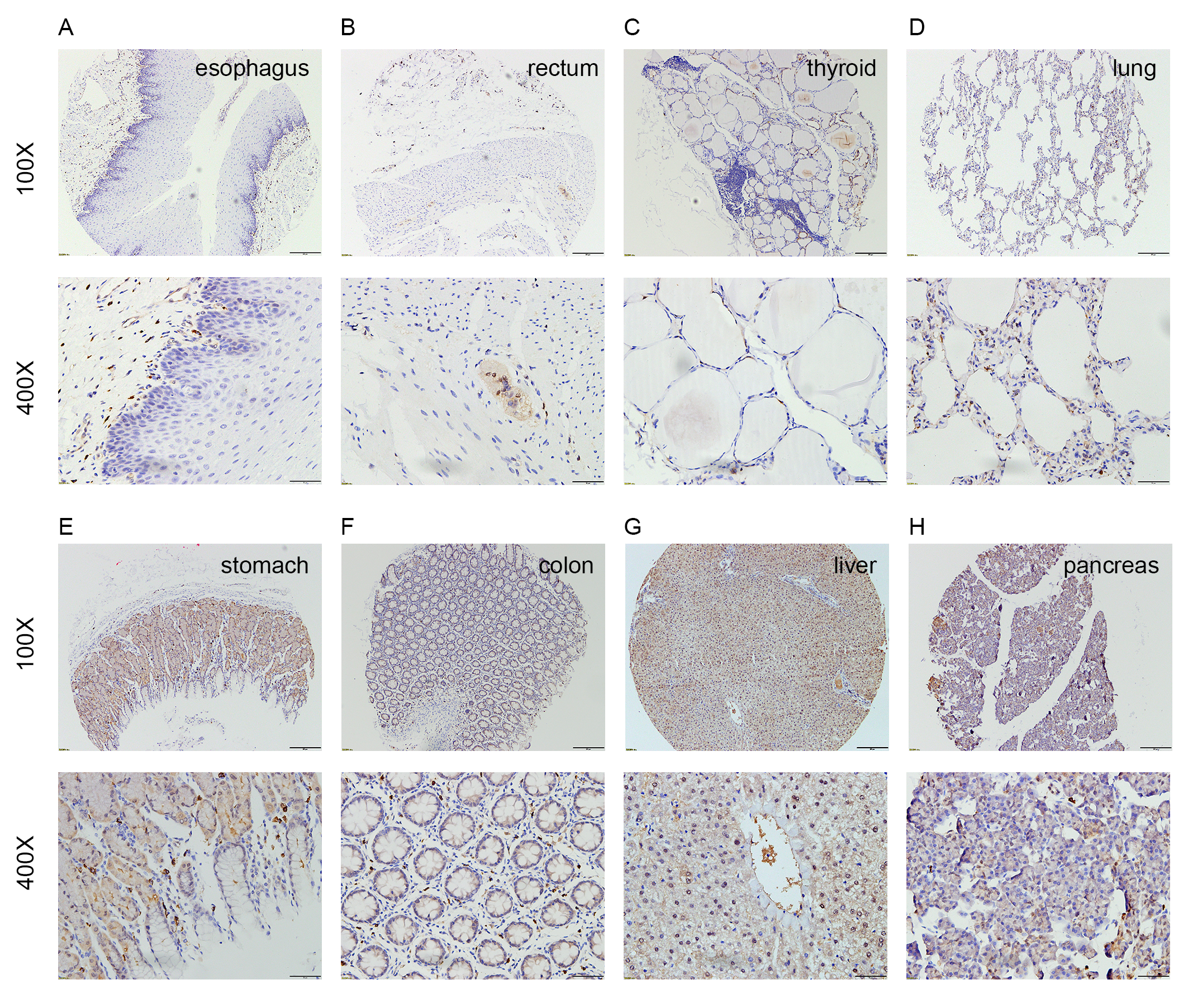

Supplement: Supplementary file 5 [file Image2.TIF]

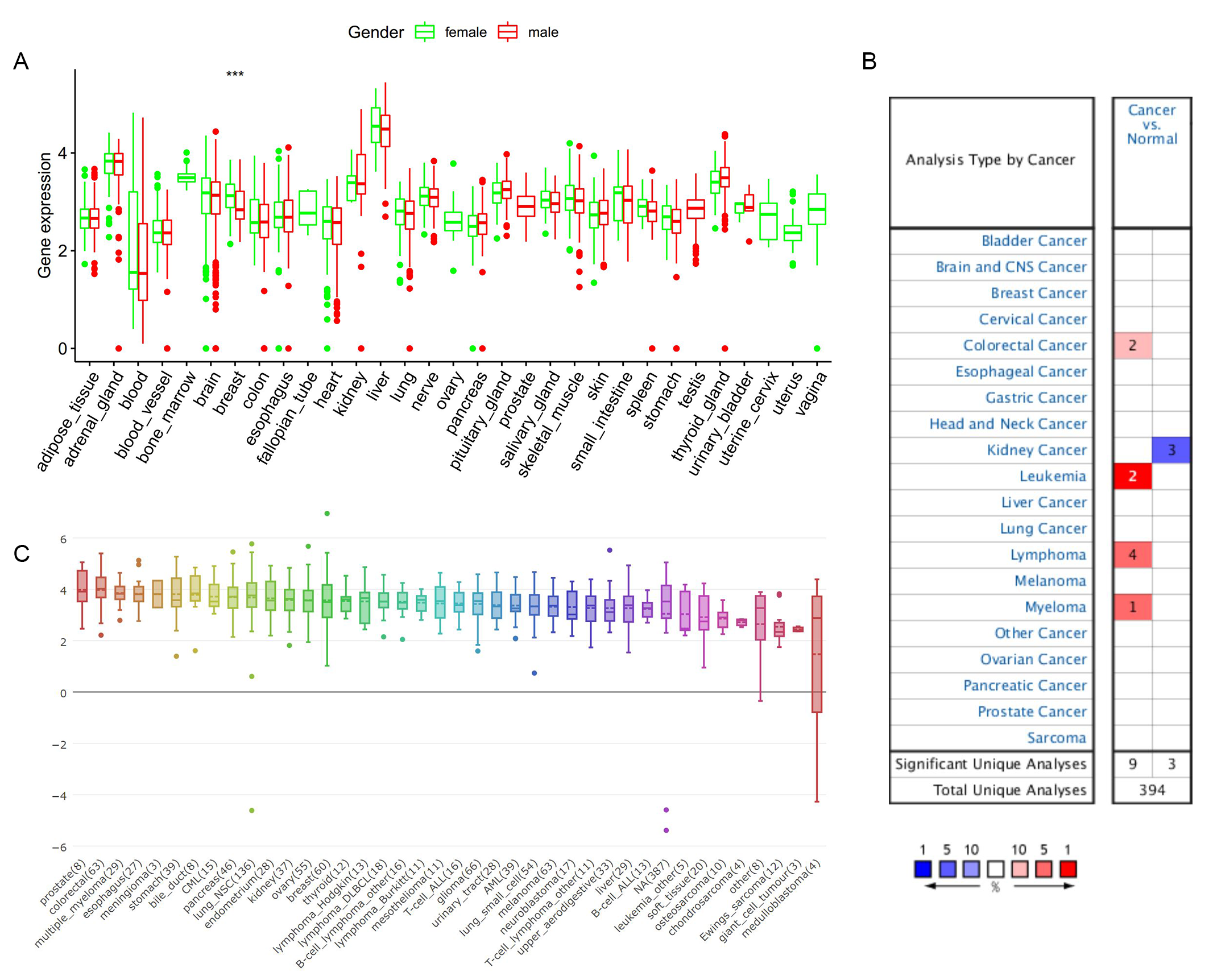

Supplement: Supplementary file 6 [file Image1.TIF]

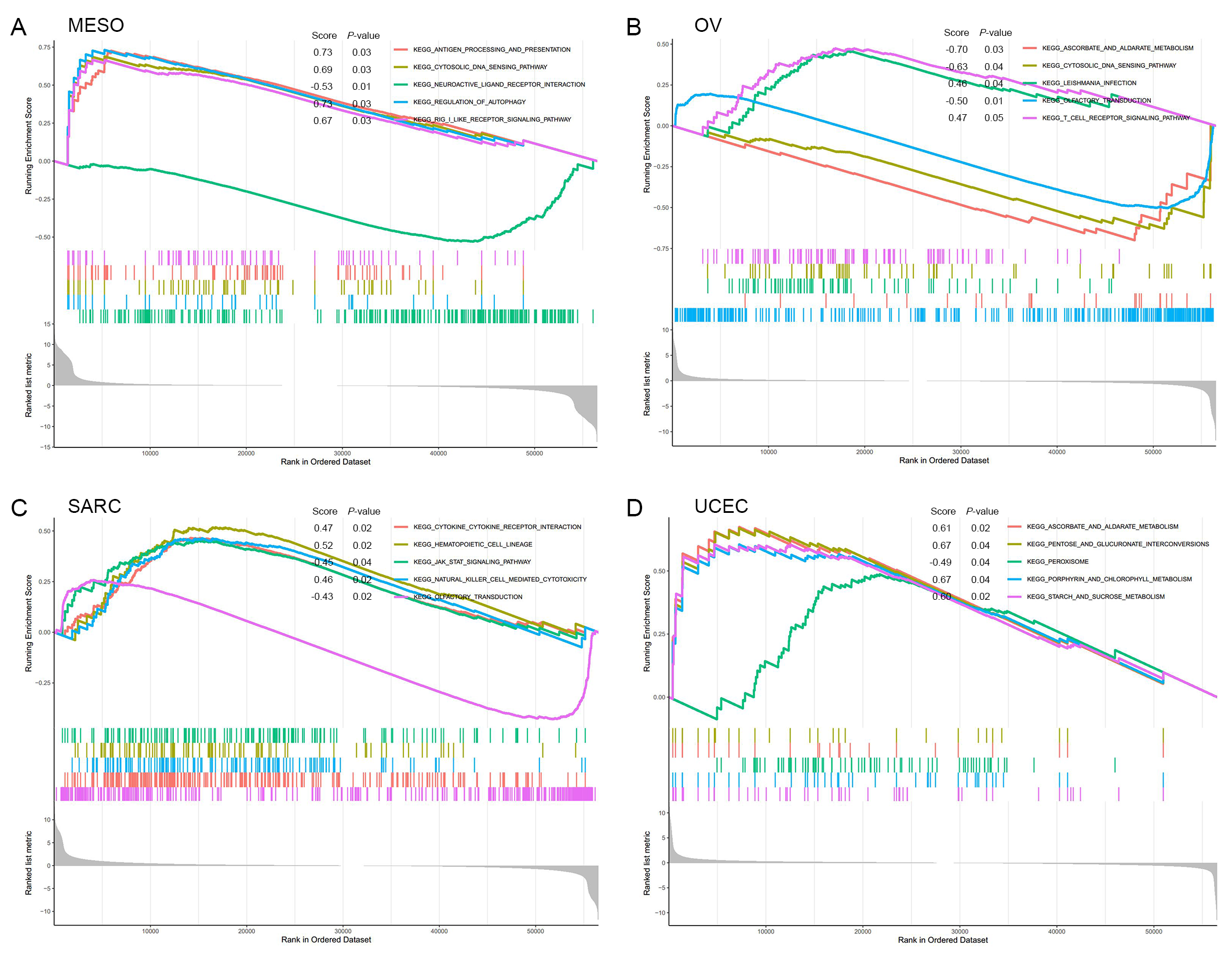

Supplement: Supplementary file 7 [file Image7.TIF]

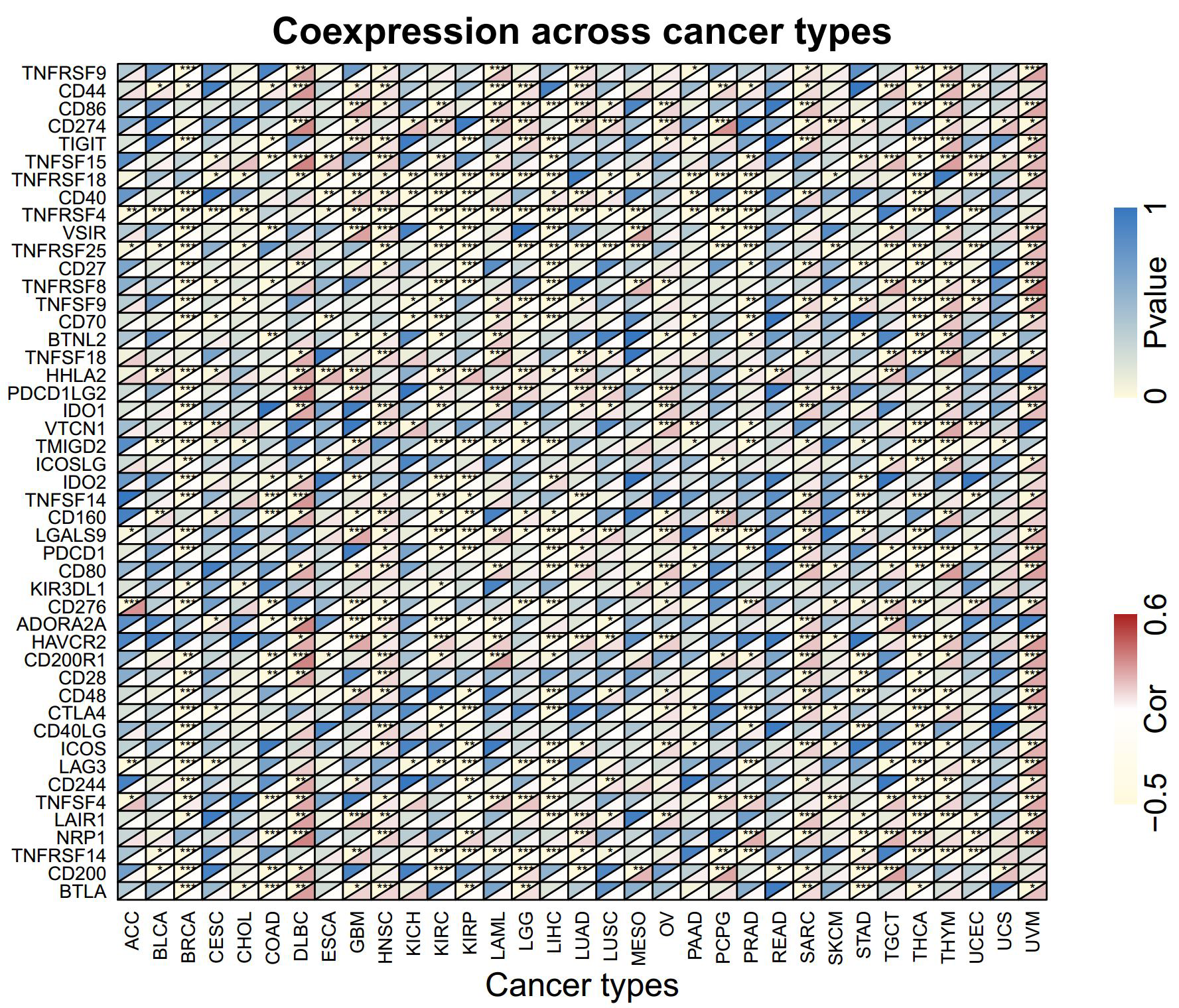

Supplement: Supplementary file 11 [file Image5.TIF]
