# Supplementary material for: Prognostic Implication and Oncogenic Role of PNPO in Pan-Cancer
Source: Front Cell Dev Biol. 2022 Jan 21;9:763674. doi: 10.3389/fcell.2021.763674 (PMC8814662; doi:10.3389/fcell.2021.763674)
Supplement: Supplementary file 2 [file Table1.DOCX]

**SUPPLEMENTARY TABLE 1** The protein expression of PNPO in pan-cancer by tissue microarray.

| Cancer type | n | PNPO expression | | *P*-value |
| --- | --- | --- | --- | --- |
|  |  | Positive | Negative |  |
| BLCA |  |  |  | **0.0030*** |
| cancer | 16 | 9 | 7 |  |
| control | 11 | 0 | 11 |  |
| BRCA |  |  |  | 0.3750 |
| cancer | 7 | 5 | 2 |  |
| control | 1 | 0 | 1 |  |
| CESC |  |  |  | **0.0002*** |
| cancer | 8 | 8 | 0 |  |
| control | 8 | 0 | 8 |  |
| COAD |  |  |  | **0.0476*** |
| cancer | 5 | 4 | 1 |  |
| control | 5 | 0 | 5 |  |
| ESCA |  |  |  | 0.4444 |
| cancer | 5 | 2 | 3 |  |
| control | 5 | 0 | 5 |  |
| HNSC |  |  |  | 1.000 |
| cancer | 3 | 2 | 1 |  |
| control | 3 | 1 | 2 |  |
| KIRC |  |  |  | NA |
| cancer | 5 | 5 | 0 |  |
| control | 5 | 5 | 0 |  |
| LIHC |  |  |  | NA |
| cancer | 5 | 5 | 0 |  |
| control | 5 | 5 | 0 |  |
| LUAD |  |  |  | **0.0152*** |
| cancer | 5 | 4 | 1 |  |
| control | 6 | 0 | 6 |  |
| LUSC |  |  |  | 0.1429 |
| cancer | 4 | 3 | 1 |  |
| control | 4 | 0 | 4 |  |
| PAAD |  |  |  | 0.2063 |
| cancer | 5 | 1 | 4 |  |
| control | 5 | 4 | 1 |  |
| READ |  |  |  | 1.0000 |
| cancer | 3 | 1 | 2 |  |
| control | 3 | 0 | 3 |  |
| STAD |  |  |  | 1.0000 |
| cancer | 5 | 1 | 4 |  |
| control | 5 | 2 | 3 |  |
| TGCT |  |  |  | 0.4000 |
| cancer | 3 | 3 | 0 |  |
| control | 3 | 1 | 2 |  |
| THCA |  |  |  | **0.0094*** |
| cancer | 12 | 11 | 1 |  |
| control | 12 | 4 | 8 |  |
| UCEC |  |  |  | 0.1515 |
| cancer | 8 | 7 | 1 |  |
| control | 3 | 1 | 2 |  |
| Penis cancer |  |  |  | 0.5238 |
| cancer | 5 | 3 | 2 |  |
| control | 5 | 1 | 4 |  |
| Ovarian cancer | 6 | 6 | 0 | NA |
| Prostate cancer | 6 | 6 | 0 | NA |
| Brain cancer | 5 | 4 | 1 | NA |
| Normal tissue | 18 | 6 | 12 | NA |
| Total | 228 | 119 | 109 | NA |

The expression of PNPO was detected by immunohistochemistry in human multiple organ tissue arrays. Fisher's exact test was applied to compare PNPO expression between cancer and adjunct tissue. n, number of cases; Positive, positive expression; Negative, negative expression. *, *P*<0.05.
