# Supplementary material for: Prognostic Implication and Oncogenic Role of PNPO in Pan-Cancer
Source: Front Cell Dev Biol. 2022 Jan 21;9:763674. doi: 10.3389/fcell.2021.763674 (PMC8814662; doi:10.3389/fcell.2021.763674)
Supplement: Supplementary file 8 [file Table2.DOCX]

**SUPPLEMENTARY TABLE 2**. Cox proportional hazards model of PNPO expression in pan-cancer.

| Cancer | OS | | PFI | | DFI | | DSS | |
| --- | --- | --- | --- | --- | --- | --- | --- | --- |
|  | *P* | HR | *P* | HR | *P* | HR | *P* | HR |
| ACC  BLCA  BRCA  CESC  CHOL  COAD  DLBC  ESCA  GBM  HNSC  KICH  KIRC  KIRP  LAML  LGG  LIHC  LUAD  LUSC  MESO  OV  PAAD  PCPG  PRAD  READ  SARC  SKCM  STAD  TGCT  THCA  THYM  UCEC  UCS  UVM | 0.117  0.589  0.098  0.368  0.991  0.916  0.316  0.927  0.900  0.125  0.539  **0.003**  0.572  **0.014**  0.138  0.238  0.757  0.326  0.050  0.418  0.350  0.792  0.056  0.369  0.701  0.827  0.122  0.257  0.502  **0.041**  0.339  0.329  0.067 | 0.680(0.420−1.102)  1.068(0.841−1.357)  1.184(0.969−1.447)  1.222(0.789−1.892)  0.996(0.476−2.084)  1.023(0.675−1.550)  1.938(0.532−7.062)  1.022(0.643−1.624)  1.027(0.675−1.564)  1.213(0.948−1.552)  1.460(0.437−4.881)  **0.680(0.528−0.876)**  0.893(0.602−1.324)  **1.752(1.121−2.739)**  0.712(0.454−1.116)  0.904(0.764−1.069)  0.955(0.714−1.277)  1.118(0.895−1.397)  0.707(0.499−1.000)  0.923(0.759−1.121)  0.780(0.463−1.313)  1.143(0.424−3.083)  3.378(0.972−11.746)  0.715(0.344−1.486)  0.930(0.641−1.349)  1.028(0.801−1.319)  0.804(0.611−1.060)  2.186(0.566−8.447)  1.403(0.522−3.771)  **2.426(1.038−5.670)**  1.144(0.868−1.507)  1.265(0.789−2.029)  2.120(0.948−4.738) | 0.903  **0.038**  0.292  0.112  0.531  0.306  0.183  0.114  0.240  0.265  0.388  **0.004**  0.268  /  0.567  0.804  0.238  0.118  0.581  0.372  0.116  0.313  0.798  0.219  0.081  0.665  0.669  0.768  0.514  0.210  0.636  0.887  **0.016** | 1.028(0.664−1.590)  **1.281(1.014−1.619)**  0.895(0.729−1.100)  1.422(0.921−2.197)  1.224(0.650−2.306)  0.841(0.604−1.172)  2.127(0.700−6.464)  0.734(0.500−1.077)  1.304(0.838−2.028)  1.157(0.896−1.494)  1.479(0.608−3.598)  **0.722(0.578−0.902)**  0.828(0.593−1.156)  /  0.895(0.614−1.307)  0.981(0.845−1.140)  0.855(0.659−1.109)  0.819(0.638−1.052)  0.900(0.618−1.310)  0.924(0.776−1.099)  0.657(0.389−1.109)  0.749(0.427−1.313)  1.052(0.711−1.557)  0.699(0.395−1.237)  0.765(0.567−1.033)  1.047(0.851−1.287)  0.938(0.699−1.258)  1.070(0.683−1.677)  1.180(0.718−1.941)  1.451(0.811−2.598)  1.058(0.838−1.336)  1.032(0.663−1.607)  **2.527(1.190−5.370)** | 0.628  **0.034**  0.741  0.086  0.835  0.622  0.911  0.189  /  0.749  0.342  0.071  0.597  /  0.252  0.719  0.803  0.966  0.666  0.631  0.589  0.474  0.839  0.746  0.092  /  0.118  0.894  0.960  /  0.621  0.349  / | 1.252(0.504−3.108)  **1.753(1.045−2.943)**  0.955(0.726−1.256)  1.858(0.916−3.772)  1.096(0.460−2.614)  0.811(0.354−1.862)  1.135(0.122−10.524)  0.595(0.274−1.291)  /  1.123(0.553−2.278)  2.272(0.418−12.339)  1.881(0.947−3.736)  0.872(0.525−1.449)  /  2.177(0.575−8.238)  0.969(0.819−1.148)  1.052(0.707−1.564)  1.009(0.677−1.503)  1.264(0.436−3.667)  0.941(0.733−1.207)  0.730(0.232−2.291)  1.783(0.367−8.663)  0.933(0.475−1.830)  1.314(0.252−6.847)  0.691(0.449−1.062)  /  0.668(0.403−1.107)  1.040(0.584−1.851)  0.983(0.495−1.952)  /  0.914(0.640−1.305)  0.632(0.242−1.651)  / | 0.198  0.331  0.344  0.438  0.979  0.143  0.817  0.389  0.885  0.266  0.308  **<0.001**  0.302  0.136  0.470  0.379  0.631  0.207  0.277  0.584  0.941  **0.002**  0.879  0.492  0.832  0.636  0.171  0.085  0.524  0.311  0.576  **0.034** | 0.719(0.434−1.189)  1.149(0.868−1.520)  0.884(0.684−1.142)  1.212(0.746−1.969)  1.009(0.514−1.980)  0.707(0.445−1.124)  1.234(0.209−7.291)  0.799(0.480−1.331)  0.965(0.596−1.563)  1.187(0.877−1.607)  1.789(0.585−5.466)  **0.575(0.442−0.749)**  0.788(0.500−1.240)  0.692(0.427−1.122)  0.927(0.755−1.138)  0.857(0.607−1.209)  1.083(0.782−1.501)  0.767(0.507−1.159)  0.887(0.715−1.101)  0.851(0.478−1.515)  1.043(0.336−3.241)  **39.31(3.72−415.1.0)**  0.925(0.337−2.538)  0.867(0.577−1.303)  0.970(0.735−1.281)  0.922(0.657−1.292)  3.319(0.595−18.521)  3.377(0.847−13.467)  1.632(0.362−7.347)  1.190(0.850−1.665)  1.139(0.722−1.797)  **2.452(1.071−5.612)** |

OS, overall survival; PFI, progression-free interval; DFI, disease-free interval; DSS, disease-specific survival. *P*, *P*-value; HR, hazard ratio.
