# Supplementary material for: Prognostic Implication and Oncogenic Role of PNPO in Pan-Cancer
Source: Front Cell Dev Biol. 2022 Jan 21;9:763674. doi: 10.3389/fcell.2021.763674 (PMC8814662; doi:10.3389/fcell.2021.763674)
Supplement: Supplementary file 9 [file Table3.DOCX]

**SUPPLEMENTARY TABLE 3**. The correlation between the mRNA expression of PNPO (222653_at) and clinical characteristics in BRCA by Kaplan-Meier plotter database.

| Gastric cancer |  | OS |  |  |  | RFS |  |
| --- | --- | --- | --- | --- | --- | --- | --- |
|  | N | HR | *P-value* |  | N | HR | *P-value* |
| **Total** | 943 | 0.66(0.50-0.87) | **0.0026** |  | 2032 | 0.67(1.58-0.79) | **3e-07** |
| **Lymph node** |  |  |  |  |  |  |  |
| Positive | 230 | 0.53(0.32-0.87) | **0.01** |  | 814 | 0.68(0.54-0.87) | **0.0019** |
| Negative | 180 | 0.34(0.14-0.80) | **0.01** |  | 574 | 0.79(0.54-1.16) | 0.23 |
| **ER** |  |  |  |  |  |  |  |
| Positive | 554 | 0.58(0.39-0.85) | **0.0044** |  | 1417 | 0.71(0.58-0.85) | **0.0004** |
| Negative | 389 | 0.81(0.55-1.18) | 0.27 |  | 615 | 0.86(0.67-1.11) | 0.25 |
| **PR** |  |  |  |  |  |  |  |
| Positive | / | / | **/** |  | 511 | 0.77(0.53-1.11) | 0.16 |
| Negative | 291 | 1.43(0.87-2.34) | 0.16 |  | 436 | 1.07(0.77-1.51) | 0.68 |
| **HER2** |  |  |  |  |  |  |  |
| Positive | 223 | 0.78(0.48-1.29) | 0.34 |  | 461 | 0.61(0.45-0.83) | **0.0015** |
| Negative | 720 | 0.64(0.47-0.89) | **0.007** |  | 1571 | 0.75(0.63-0.89) | **0.0011** |
| **Grade** |  |  |  |  |  |  |  |
| 1 | 26 | 0.24(0.02-3.30） | 0.26 |  | 113 | 0.63(0.22-1.83) | 0.39 |
| 2 | 64 | 0.55(0.18-1.75) | 0.31 |  | 243 | 0.80(0.49-1.32) | 0.39 |
| 3 | 204 | 0.44(0.26-0.75) | **0.0019** |  | 481 | 0.56(0.41-0.75) | **0.00012** |
| **StGallen** |  |  |  |  |  |  |  |
| Basal | 278 | 0.91(0.57-1.45) | 0.70 |  | 417 | 0.83(0.61-1.13) | 0.23 |
| Luminal A | 377 | 0.68(0.42-1.10) | 0.11 |  | 952 | 0.75(0.59-0.95) | **0.018** |
| Luminal B | 177 | 0.64(0.34-1.18) | 0.15 |  | 465 | 0.70(0.52-0.94) | **0.018** |

OS, overall survival; RFS, relapse-free survival. N, number; HR, hazard ratio.
