# Supplementary material for: Prognostic Implication and Oncogenic Role of PNPO in Pan-Cancer
Source: Front Cell Dev Biol. 2022 Jan 21;9:763674. doi: 10.3389/fcell.2021.763674 (PMC8814662; doi:10.3389/fcell.2021.763674)
Supplement: Supplementary file 10 [file Table4.DOCX]

**SUPPLEMENTARY TABLE 4.** Analysis of scores of immune and stromal cells with PNPO expression by ESTIMATE algorithm.

| Cancer | ImmuneScore | |  | StromalScore | |
| --- | --- | --- | --- | --- | --- |
|  | R | P |  | R | P |
| BLCA | / | / |  | -0.17 | 0.00065 |
| BRCA | -0.23 | 9.6e-15 |  | -012 | 4.12e-05 |
| CESC | / | / |  | -0.18 | 0.0016 |
| COAD | / | / |  | -0.16 | 0.00053 |
| GBM | 0.32 | 2.9e-05 |  | 0.25 | 0.0014 |
| KIRC | / | / |  | -0.18 | 3.3e-05 |
| KIRP | -0.2 | 0.00067 |  | / | / |
| LGG | / | / |  | 0.15 | 0.00072 |
| LIHC | -0.29 | 1.2e-08 |  | / | / |
| MESO | / | / |  | -0.39 | 0.00026 |
| PAAD | -0.22 | 0.0036 |  | -0.25 | 0.00071 |
| PCPG | -0.23 | 0.0017 |  | -0.24 | 0.0013 |
| PRAD | -0.19 | 2e-05 |  | / | / |
| SRAC | 0.16 | 0.0082 |  | / | / |
| STAD | -0.17 | 0.00085 |  | -0.26 | 3.9e-07 |
| TCGT | / | / |  | 0.37 | 3.3e-06 |
| THCA | -0.41 | <2.2e-16 |  | -0.36 | <2.2e-16 |
| UCEC | -0.17 | 0.00011 |  | -0.18 | 1.9e-05 |
| UVM | 0.36 | 0.00095 |  | / | / |
